# Broccoli-Derived Exosome-like Nanoparticles Alleviates Metabolic Dysfunction-Associated Steatotic Liver Disease Through Modulating the Gut–Liver Axis

**DOI:** 10.3390/nu18060953

**Published:** 2026-03-18

**Authors:** Feng Zhang, Ruolan Liu, Tongxiao Xu, Wentao Xu, Kunlun Huang, Xiaoyun He

**Affiliations:** 1 Key Laboratory of Precision Nutrition and Food Quality, College of Food Science and Nutritional Engineering, China Agricultural University, Beijing 100083, China; zhangfengyx818929@163.com (F.Z.); liuruolan6666@163.com (R.L.); tongxiao_xu23@163.com (T.X.); foodsafety@cau.edu.cn (K.H.); 2 Beijing Laboratory for Food Quality and Safety, Department of Nutrition and Health, China Agricultural University, Beijing 100191, China; xuwentao@cau.edu.cn; 3 Key Laboratory of Safety Assessment of Genetically Modified Organism (Food Safety), Ministry of Agriculture and Rural Affairs of the P.R. China, Beijing 100083, China

**Keywords:** MASLD, gut–liver axis, broccoli-derived exosome-like nanoparticles, bile secretion, hepatic inflammation

## Abstract

**Background/Objectives**: Metabolic dysfunction-associated steatohepatitis (MASLD) represents a prevalent liver disease worldwide. It is crucial to maintain the stability of the gut–liver axis in order to inhibit the advancement of MASLD. Broccoli-derived exosome-like nanoparticles (BDENs) can alleviate constipation and improve colitis. This study investigated whether BDENs possess therapeutic potential for improving induced MASLD by the gut–liver axis. **Methods**: BDENs were fractionated from fresh broccoli using differential centrifugation, and the microRNAs were identified and analyzed. 24 male C57BL/6J mice (6 weeks old) were randomized into the control group, HFD group, and BDENs group, with 8 mice per group. After 8 weeks of high-fat diet modeling, the BDENs group accepted BDENs daily oral gavage of 100 mg/kg (B.W.), while the control and HFD groups accepted 1 × PBS. Four weeks after BDENs intervention, analysis was conducted on liver injury markers, liver tissue pathology, intestinal barrier, cecal content metabolomics and fecal 16S rRNA, serum inflammatory factors, and hepatic inflammation. **Results**: BDENs identified 1659 miRNAs associated with physiological processes such as immunity, antioxidant defense, and fatty acid biosynthesis. BDENs significantly reduced weight and ALT/AST ratio (*p* < 0.05). Furthermore, BDENs attenuated hepatic histopathological damage and lipid accumulation. For the gut–liver axis, BDENs maintained intestinal barrier, regulated intestinal bile acid metabolism and restored the gut microbiota. Additionally, BDENs reduced serum LPS level (*p* < 0.01) and suppressed hepatic inflammation, including F4/80 and IL-6, IL-1β (*p* < 0.0001). **Conclusions**: Oral BDENs therapy demonstrates potential for ameliorating MASLD.

## 1. Introduction

Metabolic dysfunction-associated steatotic liver disease (MASLD) is the most prevalent type of liver disease, affecting approximately 38% of adults worldwide [1,2,3]. Concurrently, MASLD elevates the incidence of multiple pathological conditions, such as cardiovascular disease and kidney disease [4,5]. MASLD’s pathogenesis has been shown to be multifaceted, involving a number of factors including hepatic lipid toxicity, insulin resistance, endoplasmic reticulum stress, inflammatory responses and dysbiosis of the gut microbiota. Consequently, MASLD remains without universally effective treatments, the prevailing strategy being dual in nature, comprising lifestyle modifications and dietary interventions in its early phases [6]. The therapeutic options for MASLD remain extremely limited. In 2024, the U.S. FDA only authorized resmetirom for the therapy of MASH [7,8]. Therefore, novel and effective solutions require development to address MASLD.

The gut–liver axis has garnered substantial recognition since its proposal in 1998 [9]. The regulatory mechanisms of the gut–liver axis have been primarily linked to the gut microbiota and the intestinal barrier. The gut microbiota is critical for maintaining gut–liver axis homeostasis, and unhealthy diets, especially high-fat diets, can disrupt gut microbiome balance and compromise the gut barrier, thereby increasing the risk of leaky gut, leading to liver injury and inflammation [10]. Alterations in the composition of the gut microbiota have been demonstrated to influence the synthesis of diverse metabolites, including short-chain fatty acids (SCFA), bile acids (BA), and aromatic amino acids (AAA) [11]. Concurrently, dysbiosis compromises the integrity of the intestinal barrier, resulting in elevated intestinal permeability. Harmful substances are then transported via the bloodstream to the liver, thereby exacerbating liver disease. Bile acids regulate hepatic metabolism, remodel the gut microbiome or maintain the integrity of the gut barrier [12,13]. Probiotics and bioactive food components can be used to regulate gut microbiota, reduce intestinal LPS production and suppress liver inflammation through dietary supplementation [14,15].

Food-derived exosome-like nanoparticles (FDENs) represent a category of bioactive nanovesicles, with milk, fruits, and vegetables constituting plentiful sources [16,17,18]. The distinctive vesicular structure of FDENs affords them the capacity to resist gastrointestinal digestion, thereby preserving their functional activity [17,19]. It has been found that FDENs enhance intestinal and metabolic disorders by modulating the gut–liver axis. Exosome-like nanoparticles, isolated from purslane, have been shown to suppress inflammatory factor expression and maintain gut microbial diversity in colitis mice [20]. Shiitake mushroom-derived exosome-like nanoparticles modulated the gut microbiota and fecal metabolite composition in aged mice, thereby enhancing their cognitive function [21]. Furthermore, FDENs have the capacity to traverse the intestinal tract, reaching and exerting effects on other organs throughout the body. For instance, yam-derived exosome-like nanoparticles have been observed to promote osteoblast differentiation and enhance bone growth in osteoporotic mice [22]. Garlic-derived nanoparticles suppressed hepatic autophagy and inflammatory infiltration in murine models of acute liver injury [23].

As of yet, the study of FDENs remains in its infancy. The primary routes of administration for FDENs comprise oral and intravenous methods, both of which exhibit favourable safety profiles. For instance, following the administration of both milk-derived and plant-derived FDENs via two routes, no immune reactions or liver function impairment were observed in the animals [24,25,26,27]. However, it is important to note that at exceedingly high doses, adverse effects may emerge [28,29]. A number of FDENs have been registered for clinical-stage trials, but none have yet been approved for the treatment of the disease. FDENs derived from grapes have been the subject of clinical trials in order to evaluate their potential to prevent chemotherapy- and radiotherapy-induced oral mucositis in patients with head and neck cancer [30]. FDENs obtained from aloe vera and ginger have also been clinically employed to alleviate the symptoms of polycystic ovary syndrome [30]. Consequently, there is an ongoing development of FDENs as innovative medical oral nano-products, particularly within the domains of intestinal diseases and gut–liver axis-related metabolic disorders.

Broccoli (*Brassica oleracea* L. ssp. *italica*), a widely consumed vegetable, contains various bioactive compounds, such as glucosinolates and phenolic compounds, and exhibits lipid-lowering, anti-cancer, and anti-inflammatory functions [31,32,33]. Broccoli-derived exosome-like nanoparticles (BDENs) can alleviate constipation in mice, improve intestinal damage in DSS-induced colitis models, and exhibit anti-pancreatic cancer effects [34,35,36]. However, whether BDENs possess hepatoprotective effects remains unexplored. Therefore, this study established a mouse model of MASLD through the feeding of mice a high-fat diet, liver injury markers (ALT/AST) were measured, and liver histology was observed to evaluate the efficacy of BDENs in improving MASLD. The protective effects of BDENs on intestinal tissue were assessed by examining intestinal pathology and intestinal barrier markers (MUC2 and Claudin-1). In addition, metabolomics analysis of cecal contents and fecal 16S rRNA sequencing were performed, alongside detection of serum inflammatory markers (LPS, IL-6, IL-1β) and hepatic inflammatory indicators (IL-6, TNF-α, IL-1β, F4/80) to investigate whether BDENs protect the liver via the gut–liver axis.

## 2. Materials and Methods

### 2.1. Preparation and Evaluation of BDENs

Broccoli was procured from a local supermarket, and the extraction method was conducted as outlined previously [35,36]. Firstly, 200 g of broccoli was weighed, washed twice with sterile water, and blended with 300 mL of 4 °C 1 × PBS for 10 min in a blender at a speed of 15,000 r/min with intervals of 30 s on and 15 s off. Broccoli juice was then filtered using cheesecloth, after which the residual pulp was disposed of. The collected filtrates were centrifuged at 4 °C for 1000× *g* (10 min), 3000× *g* (20 min), and 10,000× *g* (60 min) (Eppendorf, Hamburg, Germany), respectively. The supernatants were collected from each centrifugation step to eliminate fibers and cellular debris. The collected material then undergoes filtration with a 0.45-micron filter to exclude large particles, followed by ultracentrifugation at 150,000× *g* at 4 °C for 2 h (32Ti, Beckman Coulter, Pasadena, CA, USA). The supernatant was discarded, and 1 × PBS was added and centrifuged once more. The resultant pellet was identified as BDENs. Three replicate extractions were performed using broccoli from three different batches. BDENs were resuspended in 1 × PBS, and their protein concentration was assayed using a commercial kit (Beyotime Biotechnology, Shanghai, China). BDENs were then stored at −80 °C.

The purity of BDENs was assessed using dynamic light scattering (DLS) and transmission electron microscopy (TEM). The particle size (nm) and zeta potential (mV) of BDENs were evaluated utilizing DLS technology with a Malvern nanoparticle size analyzer (Malvern, UK) [37]. The morphological features of BDENs were observed using a TEM (HT7700, Hitachi, Tokyo, Japan). MicroRNAs (miRNAs) analysis of BDENs was executed by Shanghai Majorbio Biopharmaceutical Technology Co., Ltd. (Shanghai, China).

### 2.2. Animal Experiments

The experimental design was permitted by the Animal Care and Use Committee of China Agricultural University (No. Aw62214202-5-08). The study utilized male C57 mice, aged six weeks, which procured from Beijing Charles River Laboratories and subsequently housed in an SPF facility (22 ± 2 °C, 40–60% humidity, 12 h light/dark cycle, from 7 a.m. to 7 p.m.). Animals were housed in individually ventilated cages made of polycarbonate, with four animals per cage, measuring 375 × 160 × 180 mm. Bedding was sterilized with cork shavings; enrichment items were chewable wood blocks, replaced weekly. Animals had free access to water and feed. Based on the foundation of our laboratory’s previous research [38,39], 24 mice were randomly assigned to a control group (*n* = 8) and a high-fat diet modeling group. The control group consumed maintenance chow (Beijing Keao Xieli Feed Co., Ltd., Beijing, China) throughout the study. The model group ingested a 60% fat diet (Research Diets, New Brunswick, NJ, USA) for eight weeks. Following the 8-week modeling period, mice were re-randomized into two subgroups (*n* = 8): the HFD group and the BDENs group. The HFD group was administered sterile 1 × PBS by daily gavage at a volume of 100 μL/10 g body weight (B.W.). The BDENs group was given 100 mg/kg of BDENs (protein concentration) by daily gavage at 10:00 a.m., with a gavage volume of 100 μL per 10 g (B.W.).

The BDENs intervention cycle was conducted over a period of four weeks, body weight was measured every week, and food intake of the mice was recorded every three days. Two days before the experiment concluded, fresh mouse feces were collected using sterile instruments and promptly transferred to liquid nitrogen for cryopreservation. At the conclusion of the animal experiment, mice were subjected to a 6 h fast. Mice were anesthetized via intraperitoneal injection of 1% sodium pentobarbital (50 mg/kg), and whole blood samples were collected through orbital puncture. Subsequently, euthanasia was performed by cervical dislocation under deep anesthesia. Serum, liver tissue, subcutaneous fat (Sub), epididymal fat (EP), spleen, kidney, cecal contents, and colon tissue were collected from the mice. A small portion of each organ tissue was immersed in tissue fixative, whereas the majority was cryopreserved at −80 °C. Histological section evaluation and biochemical indicator testing were both performed independently by two researchers who were unaware of the experimental groupings.

### 2.3. Serum Biochemical Analysis

Serum biochemical markers were measured using an Indiko™ Plus Chemistry Instrument (Thermo, Waltham, MA, USA), including ALT, AST, HDL, and LDL.

### 2.4. Pathological Analysis

The liver and colon tissues of the mice were fixed in 4% paraformaldehyde, paraffin-embedded, sectioned, and H&E staining was performed to evaluate the pathological condition. Liver sections were stained with Oil Red O to assess steatosis. For immunohistochemistry (IHC), the primary antibodies used were MUC2 (ab272692, Abcam, Cambridge, UK) and Claudin-1 (ab211737, Abcam, Cambridge, UK). For immunofluorescence, the primary antibody used was theF4/80 (ab300421, Abcam, Cambridge, UK).

### 2.5. Metabolomics

Mouse cecum samples underwent preprocessing and were subsequently analyzed for metabolomics at Majorbio Bio-Pharm Technology Co., Ltd. (Shanghai, China). For specific methodology, see the Supplementary Materials.

### 2.6. 16S rRNA Sequencing

Fecal samples from mice were subjected to 16S rRNA sequencing analysis at Majorbio Bio-Pharm Technology Co., Ltd. (Shanghai, China). For specific methodology, see the Supplementary Materials.

### 2.7. ELISA

Serum levels of LPS, IL-6, and IL-1β were measured using ELISA kits (Cusabio, Wuhan, China) according to the instructions.

### 2.8. qRT-PCR Analysis

RNA separation was accomplished utilizing TRIzol reagent (Invitrogen, Carlsbad, CA, USA). The purity of the extracted RNA was determined using a NanoDrop 2000 spectrophotometer (Thermo), with the A260/A280 ratio of all samples maintained between 1.9 and 2.1. An equal quantity of RNA (1 μg) was subjected to reverse transcription. The reverse transcription of RNA into cDNA was accomplished through the utilization of a reverse commercial kit (TransGen Biotech, Beijing, China). The cDNA was subjected to qRT-PCR using a fluorescent commercial kit (TIANGEN, Beijing, China). Primers were in Table S1.

### 2.9. Statistical Analysis

According to the Shapiro–Wilk test, the data in each group follow a normal distribution (*p* > 0.05). All results are represented as mean ± SD. Experimental results were organized using Office 2021 Excel and analyzed by GraphPad Prism 9.5. One-way ANOVA combined with Tukey’s multiple comparison test was conducted to ascertain the significance of the results. *p* < 0.05 was considered statistically significant.

## 3. Results

### 3.1. Isolation and Characterization of BDENs

**Figure 1:**
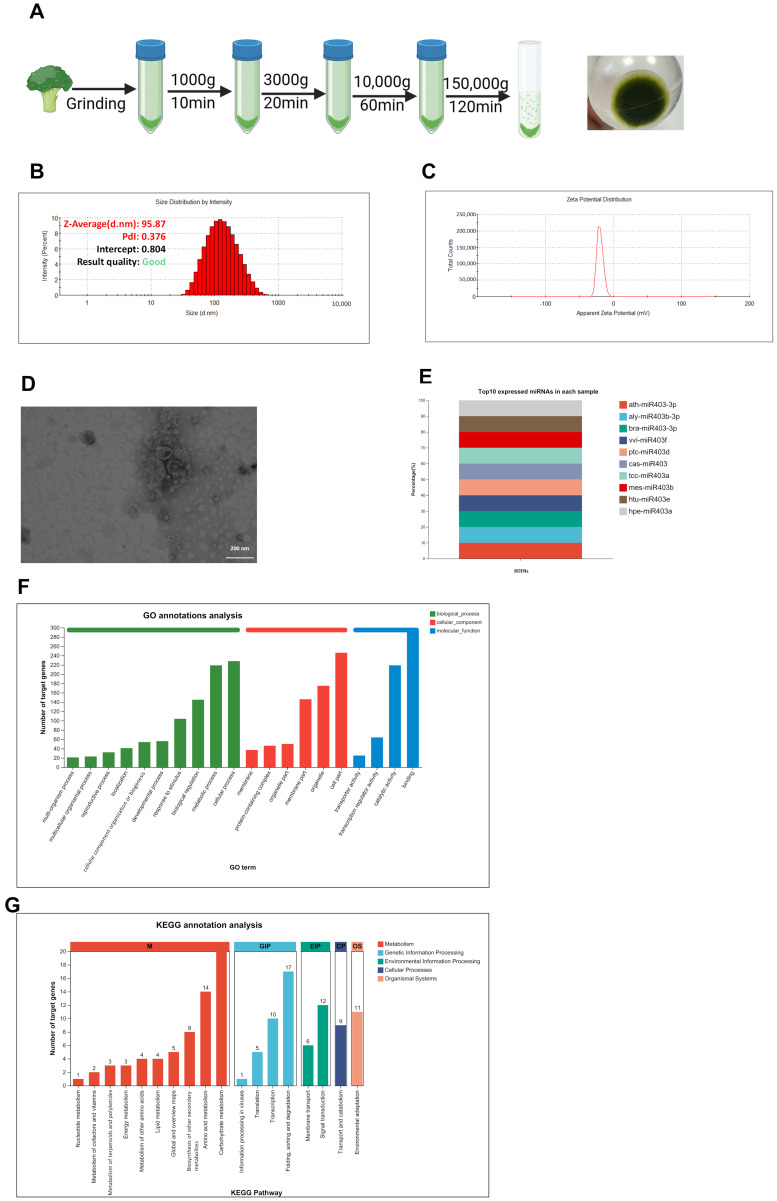
Isolation and characterization of BDENs. (**A**) Schematic illustration of BDENs isolation process, created by Biorender. (**B**) Size of BDENs detected by dynamic light scattering (DLS). (**C**) Zeta potential of BDENs detected by DLS. (**D**) Representative image of BDENs detected by TEM. (**E**) The 10 most highly expressed miRNAs in BDENs. (**F**) GO annotations analysis of BDENs miRNA. (**G**) KEGG annotations analysis of BDENs miRNA.

BDENs were obtained through the process of gradient centrifugation at different rotational speeds, as illustrated in Figure 1A. DLS detection confirmed the particle size of BDENs was approximately 95 nm (Figure 1B). The zeta potential of BDENs was about −30 mV (Figure 1C). The results of three independent replicate experiments for protein concentration, particle size, and potential of BDENs are shown in Supplementary Figure S1A and Table S2, indicating that the extraction and characterization of BDENs exhibit good reproducibility. The TEM observation demonstrated that the isolated BDENs manifested a distinctive cup-shaped morphology, consistent with the characteristics of exosomal structures (Figure 1D). Protein concentration was used to calculate the output of BDENs, which was about 0.3 mg/g.

### 3.2. miRNA Analysis of BDENs

In this study, miRNAs in BDENs were identified, analyzed for expression levels, and underwent target gene assessment. 1659 miRNAs were identified, including both known and newly predicted species. The top ten expressed miRNAs in BDENs were illustrated in Figure 1E. GO analysis revealed that the top 50 most expressed miRNAs primarily participated in 15 biological processes (BP), such as metabolic process and immune system processes; 10 cellular components (CC), such as membrane part; and 9 molecular functions (MF), including antioxidant potency and transcription factor potency (Figure 1F). KEGG analysis revealed that miRNAs in BDENs primarily participate in 42 metabolisms, including fatty acid biosynthesis and pyruvate biochemical processes; 11 genetic information processing pathways, such as SNARE interactions in vesicular transport; 4 environmental information processing pathways; 4 cellular processes; and 2 organismal systems (Figure 1G). The results of the analysis of miRNA indicated that BDENs are associated with processes such as lipid metabolism, immunity, and antioxidant defense. This suggested that BDENs have the potential to ameliorate MASLD.

### 3.3. BDENs Decreased Body Weight and Alleviated Hepatic Injury in HFD Mice

**Figure 2:**
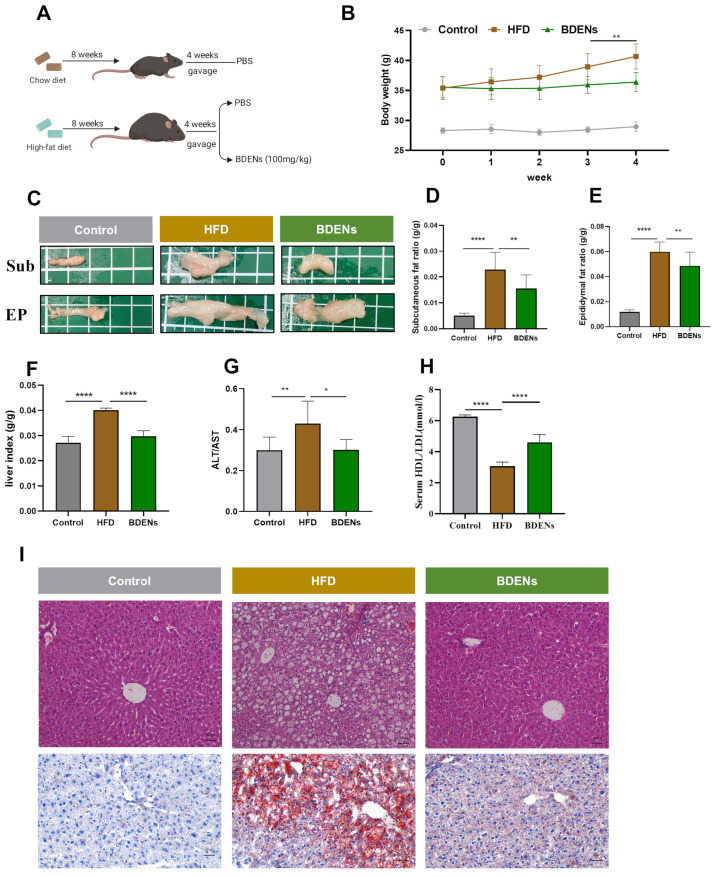
BDENs decreased body weight and alleviated hepatic injury. (**A**) Schematic illustration of the animal experiment, created by Biorender. (**B**) Changes in body weight under BDENs intervention. (**C**) Representative images of epididymal fat and subcutaneous fat. (**D**,**E**) Subcutaneous fat ratio and epididymal fat ratio. (**F**) Liver index (liver weights/body weights). (**G**) ALT/AST. (**H**) HDL/LDL. (**I**) Liver H&E staining and oil red O staining. (*n* = 6 per group) * *p* < 0.05, ** *p* < 0.01, **** *p* < 0.0001, compared to the HFD group.

The experimental design was illustrated in Figure 2A. Following the completion of the four-week BDENs supplement, BDENs group mice exhibited a significant decline in body weight (Figure 2B). There was no significant difference in food intake between the BDENs group and the HFD group (Figure S1B). Furthermore, a high-fat diet significantly promoted white fat formation in mice, comprising both subcutaneous fat and epididymal fat (Figure 2C–E). Notably, BDENs dramatically diminished production of Sub and EP. No significant abnormalities were observed in the pathological sections of the spleen and kidneys across all groups (Figure S1C). The above results revealed that BDENs can alleviate obesity in mice.

Next, we further studied the protective function of BDENs on the liver. As demonstrated in Figure 2F, the liver organ index in the HFD group rose, while the BDENs group significantly reversed this trend. Liver injury markers ALT and AST results showed that BDENs intervention markedly reduced the ALT/AST ratio (Figure 2G). HDL and LDL provide an indication of fatty accumulation in hepatic tissue, and metabolic abnormalities are often characterized by decreased HDL/LDL ratio. BDENs significantly increased the HDL/LDL ratio, indicating the restoration of abnormal lipid metabolism in mouse livers (Figure 2H). Furthermore, pathological analysis discovered distinct pathological features in the HFD group, including marked hepatic steatosis and balloon-like vacuoles (Figure 2I). Treatment with BDENs effectively suppressed these hepatic pathological damages.

### 3.4. BDENs Ameliorated Gut Barrier and Intestinal Metabolism in HFD Mice

**Figure 3:**
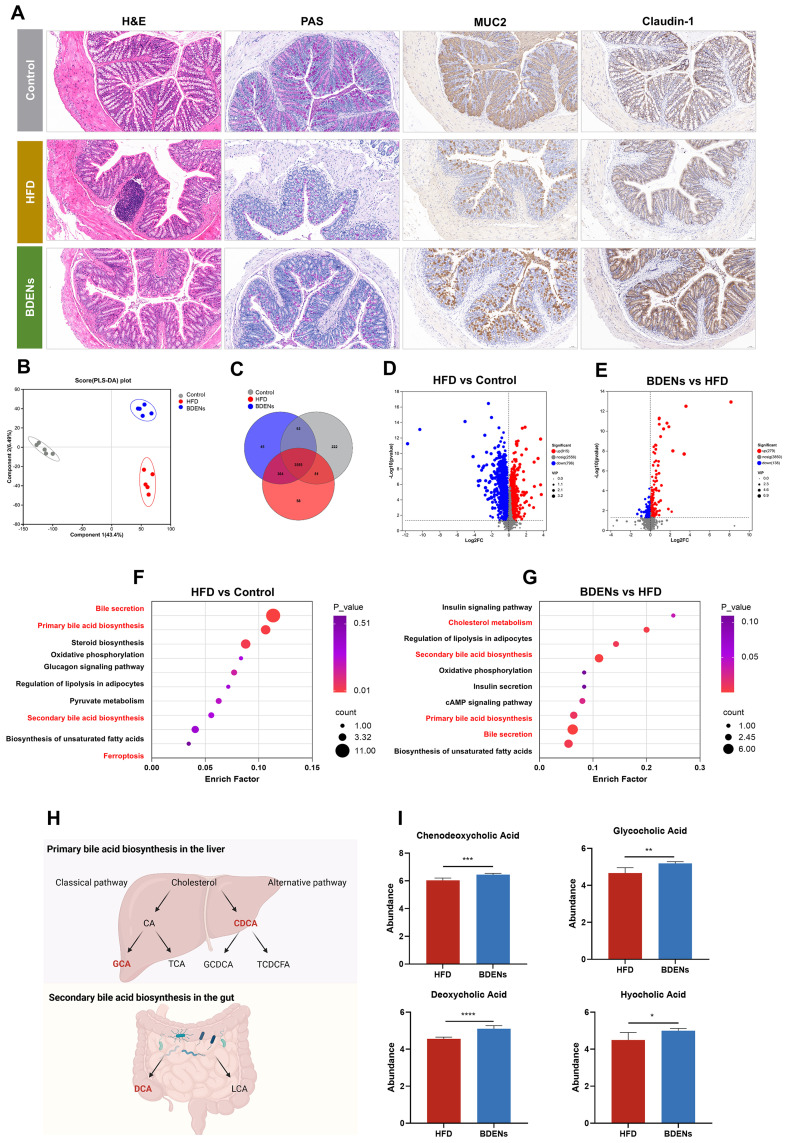
BDENs ameliorated gut barrier and intestinal metabolism. (**A**) PAS staining and H&E staining of the gut, IHC staining of the gut MUC2 and Claudin-1. (**B**) PLS-DA analysis of metabolites. (**C**) Venn diagrams for different groups of metabolites. (**D**,**E**) Volcano plot. (**F**,**G**) KEGG enrichment analysis of differential metabolites. (**H**) Schematic diagram of bile acid metabolism, created through Biorender. (**I**) Relative abundance of bile acid metabolites. (*n* = 5 per group). * *p* < 0.05, ** *p* < 0.01, *** *p* < 0.001, **** *p* < 0.0001.

Histological analysis demonstrated that 100 mg/kg BDENs reduced high-fat diet-induced colonic epithelium disruption (Figure 3A). The protein expression of gut barrier-related genes MUC2 and Claudin-1 also increased significantly after 100 mg/kg BDENs intervention, illustrating that BDENs can restore high-fat diet-induced intestinal mucosal barrier damage.

For further elucidation of the hepatoprotective mechanism of BDENs, we performed metabolomic analysis of mouse cecum contents. PLS-DA exhibited notable separation among the three groups, demonstrating that the high-fat diet significantly altered intestinal metabolism (Figure 3B). Moreover, the Venn diagram illustrated a total of 3555 metabolites among the three groups (Figure 3C). Based on further analysis, HFD elevated 915 metabolites and reduced 796 metabolites in comparison to the control group, while BDENs elevated 279 metabolites and reduced 138 metabolites compared to the HFD group (Figure 3D,E). Furthermore, KEGG enrichment analysis revealed that HFD disrupted Bile secretion, bile acid biosynthesis, and Ferroptosis (Figure 3F). BDENs regulated intestinal metabolism mainly through Cholesterol metabolism, Bile secretion, Primary bile acid biosynthesis and Secondary bile acid biosynthesis (Figure 3G). These results indicated that BDENs could modulate the intestinal metabolic disorders induced by a high-fat diet.

The metabolization process of bile acids mainly includes the classical pathway and the alternative pathway, and the specific metabolic process is displayed in Figure 3H. In the BDENs group, chenodeoxycholic acid (CDCA), glycocholic acid (GCA) and deoxycholic acid (DCA) were increased in abundance (Figure 3I).

### 3.5. BDENs Ameliorated Gut Microbial Disorders in HFD Mice

**Figure 4:**
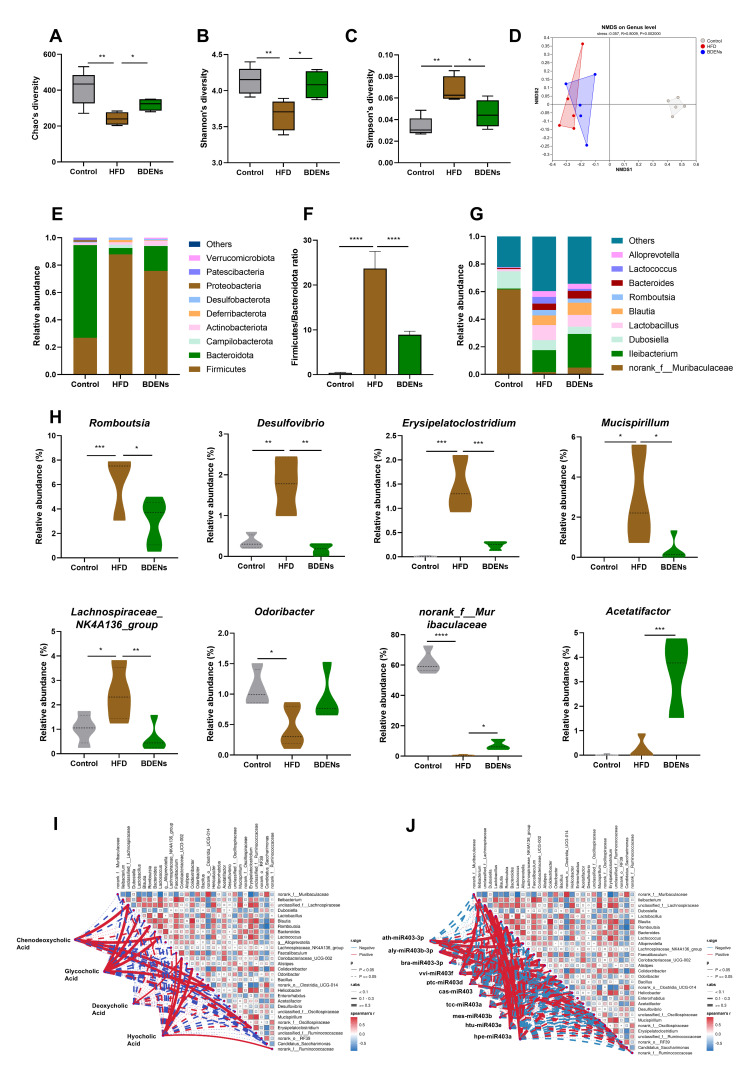
BDENs ameliorated gut microbial disorders in HFD mice. (**A**–**C**) Chao index, Shannon index and Simpson index of intestinal microbiota. (**D**) NMDS (Non-metric Multi-Dimensional Scaling) of intestinal microbiome. (**E**) Intestinal microbiome at the phylum level. (**F**) Firmicutes/Bacteroidota ratios of the intestinal microbiome. (**G**) Intestinal microbiome at the genus level. (**H**) Beneficial microbe and harmful microbe. (**I**,**J**) Spearman correlation coefficient between the genus-level intestinal microbes and the restored metabolites, between the genus-level intestinal microbes and the top 10 expressed miRNAs in BDENs. (*n* = 5 per group). * *p* < 0.05, ** *p* < 0.01, *** *p* < 0.001, **** *p* < 0.0001.

To explore whether BDENs enhance hepatoprotective effects by regulating gut microbes, we evaluated the composition of the gut microbiota. As shown in Figure 4A–C, the α-diversity analysis revealed a noticeable decline in Chao1 and Shannon indices and a marked rise in the Simpson index in the HFD group, which confirmed a significant decrease in diversity of the microbiota in the HFD group. Furthermore, the BDENs intervention restored the α-diversity index, indicating that BDENs have a protective effect on microbiota diversity. In addition, NMDS analysis showed that BDEN treatment altered the gut microbial structure of MASLD mice to more closely resemble that of controls (Figure 4D).

A high-fat diet notably modifies the phylum-level composition of gut microbiota, as evidenced by a rise in relative abundance of Campilobacterota, Deferribacterota, and Desulfobacterota in the HFD group (Figure 4E). Following BDENs administration, the abundances of Bacteroidota, Actinobacteria, and Verrucomicrobiota elevated, whereas those of Firmicutes and Deferribacterota diminished. As shown in Figure 4F, F/B ratio rose notably in the HFD group and fell significantly in the BDENs group. BDENs markedly raised the abundance of norank_f__Muribaculaceae, Acetatifactor, Odoribacter, and markedly lowered the abundance of Romboutsia, Desulfovibrio, Erysipelatoclostridium, Mucispirillum, Lachnospiraceae_NK4A136_group (Figure 4G,H). In general, BDENs protected the biodiversity of gut microbiota.

Spearman correlation analyses between differential gut microbes and intestinal metabolites were performed to further investigate the hepatoprotective effects of BDENs (Figure 4I). Both intestinal CDCA levels and GCA levels were substantially positively correlated with unclassified_f__Lachnospiraceae, Lactococcus, and Helicobac-ter (*p* < 0.05, r ≥ 0.3), and negatively correlated with Ileibacterium, Dubosiella, Desulfovibrio, and Lachnospiraceae_NK4A136_group (*p* > 0.05, 0.1 < r < 0.3). In addition, DCA levels were positively correlated with Acetatifactor and Helicobacter (*p* > 0.05, 0.1 < r < 0.3), and Coriobacteriaceae_UCG-002 (*p* < 0.05, 0.1 < r < 0.3).

Furthermore, Spearman’s correlations were analyzed between the top 10 expressed miRNAs in BDENs and gut microbiota (Figure 4J). A significant positive correlation was observed between ath-miR403-3p and Bacteroides, Alloprevotella, Alistipes, and unclassified_f__Ruminococcaceae (*p* < 0.05, r ≥ 0.3). Similarly, a significant positive correlation was identified between aly-miR403b-3p and Erysipelatoclostridium (*p* < 0.05, r ≥ 0.3). Additionally, vvi-miR403f showed significant positive correlations with Ileibacterium, Faecalibaculum, and Bacillus (*p* < 0.05, r ≥ 0.3). In the context of the study, significant positive correlations were identified between ptc-miR403d and Erysipelatoclostridium (*p* < 0.05, r ≥ 0.3). Furthermore, Htu-miR403e exhibited notable positive associations with norank_f__Muribaculaceae, unclassified_f__Lachnospiraceae, Dubosiella, Lactobacillus, and Romboutsia (*p* < 0.05, r ≥ 0.3).

### 3.6. BDENs Ameliorated Liver Inflammation via the Gut-Liver Axis

**Figure 5:**
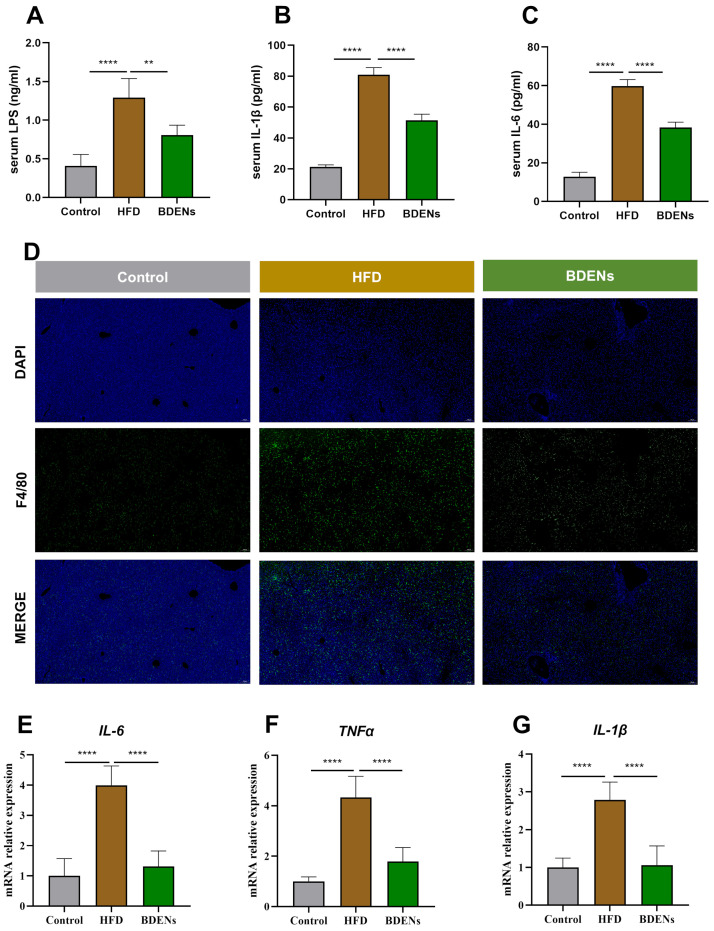
BDENs ameliorated liver inflammation via the gut–liver axis. (**A**–**C**) Serum LPS, IL-6, IL-1β. (**D**) F4/80 immunofluorescence image of the liver. (**E**–**G**) The mRNA relative expression of IL-6, TNF-α, and IL-1β in the liver. (*n* = 5 per group). ** *p* < 0.01, **** *p* < 0.0001.

As shown in Figure 5A, oral administration of BDENs significantly reduced serum LPS levels. Serum IL-6 and IL-1β also decreased markedly (Figure 5B,C). Immunofluorescence staining for F4/80 in the liver revealed a significant increase in F4/80-positive cells in the HFD group, which was markedly reduced following BDENs intervention (Figure 5D). Following BDENs treatment, the expression of IL-6, TNF-α, and IL-1β in hepatic tissue was significantly suppressed (Figure 5E–G). Collectively, these findings indicated that BDENs alleviate MASLD by repairing the intestinal barrier and reducing LPS translocation from the gut to the liver, thereby attenuating hepatic inflammation.

## 4. Discussion

Given the propensity of MASLD to progress to severe liver diseases, such as cirrhosis and hepatocellular carcinoma, the implementation of lifestyle interventions in the early stages of the disease can help to prevent its progression. In recent years, novel bioactive natural compounds have garnered increasing attention for their potential in the prevention and treatment of liver diseases. Curcumin prevents and alleviates MASLD primarily by protecting the liver through reducing hepatic steatosis and suppressing inflammation [40]. Resveratrol has the capacity to regulate hepatic lipid metabolism and inhibit oxidative stress, thereby alleviating MASLD [41]. In this study, broccoli-derived exosome-like nanoparticles (BDENs) showed potential for ameliorating MASLD.

The separation methods for food-derived exosome-like nanoparticles (FDENs) are typically determined based on the food source and the characteristics of the FDENs themselves. In order to achieve the objectives of the present experiment, a method combining differential centrifugation with ultracentrifugation was employed to extract BDENs. The extraction procedure and its reproducibility were validated through repeated centrifugation trials performed at least three times. This is similar to the extraction methods used for many FDENs derived from fruits and vegetables [42]. Characterization results of BDENs indicate a size of approximately 95 nm and a zeta potential of about -30 mV, exhibiting a distinctive cup-shaped morphology consistent with the typical characterization features of FDENs [43]. Research findings have identified that intact membrane structures are fundamental to the efficacy of FDENs, and smaller particle sizes (<200 nm) enable FDENs to better withstand the gastrointestinal environment [17,19]. Negative charges may enhance the uptake of FDENs by intestinal cells, thereby increasing the efficacy of FDENs [44].

Food-derived exosome-like nanoparticles (FDENs) exert multiple bioactive roles, like anti-inflammatory, anti-cancer, and anti-osteoporosis effects [45,46,47]. However, only a limited number of FDENs have demonstrated a positive effect on MASLD. Blueberry-derived nanoparticles (BELNs) alleviated oxidative stress and mitigated MASLD [48]. Garlic-derived exosomal nanoparticles (GaELNs) have been demonstrated to suppress systemic inflammatory activity via the gut–brain axis [49]. Oral administration of BDENs was observed to maintain the intestinal barrier, regulate gut metabolites and the composition of the gut microbiota, and alleviate liver inflammation. Consequently, BDENs have the potential to exert hepatoprotective effects through the gut–liver axis.

Cholesterol, as an integral constituent of cell membranes, is involved in various regulatory roles in cellular life activities. The liver is the main organ for cholesterol metabolism, and maintaining cholesterol homeostasis can effectively inhibit the progression of MASLD. One pivotal mechanism is the metabolism of cholesterol into bile acids and their secretion into the intestines for elimination [50]. Bile acids are initially produced in the liver and undergo secondary metabolism within the intestine. Notably, a portion of bile acids in the intestine is reabsorbed back into the liver [51]. Thus, bile acids serve as one of the pivotal links between the intestines and the liver. In addition, bile acids suppress the colonization of harmful bacteria and sustain the integrity of the gut barrier, consequently reducing the transport of hazardous substances into the liver and attenuating MASLD [10].

An unhealthy diet can disrupt the gut microbiota and damage the intestinal barrier. The amount of microbial metabolites translocated to the liver increases, aggravating the metabolic abnormality and inflammatory response of the liver, and ultimately leads to MASLD [52]. This study observed that BDENs effectively repaired the intestinal mucosa and inhibited hepatic inflammation induced by harmful substances. norank_f__Muribaculaceae enhances the integrity of gut barrier in models such as colitis and obesity [53,54]. Romboutsia is considered to be strongly associated with intestinal injury and intestinal inflammation [55]. Desulfovibrio and Erysipelatoclostridium, as pathogenic bacteria, overproliferation in the intestine will lead to alteration of intestinal permeability and harmful substances, such as endotoxin, entering the liver via the gut–liver axis to induce liver inflammation [56].

However, other mechanisms independent of microorganisms and gut metabolites also alleviate MASLD. In our study, BDENs also diminished white fat deposition, suggesting that BDENs may lower the influx of fatty acids into the liver following lipolysis.

The analysis of MicroRNAs in BDENs can only indicate that BDENs have the potential to regulate MASLD; the specific substances in BDENs that play a role in relieving MASLD remain to be elucidated. Future research needs to use omics technologies to further identify key lipids, proteins, or secondary metabolites in BDENs and to verify their specific mechanisms of action. Although our data provide preliminary evidence of safety, further large-scale dose safety validation and additional studies on toxicities such as genotoxicity and immunotoxicity are still required. Moreover, although experimental results indicate significant differences in key indicators, the limited number of animals and short intervention period may restrict our ability to conduct in-depth differential analyses based on variables such as gender and age. Future studies with larger sample sizes and longer intervention periods are needed to validate the findings of this research.

## 5. Conclusions

In summary, this study suggests that BDENs may possess the potential to alleviate hepatic steatosis and injury while regulating gut metabolites and microbiota. However, the association between BDENs and changes in gut metabolites or alterations in gut microbial genera remains unclear. The present findings offer only a suggestion that BDENs may alleviate the MASLD mouse model. Future studies should explore the translational prospect of BDENs in more humanized models or clinical trials.

## Data Availability

The original contributions presented in this study are included in the article/Supplementary Material. Further inquiries can be directed to the corresponding author.

## Supplementary Materials

The following supporting information can be downloaded at: https://www.mdpi.com/article/10.3390/nu18060953/s1, Figure S1: Characterization of BDENs and Assessment of BDEN Side Effects. (A) Size and zeta potential of BDENs detected by DLS. (B) Changes in food intake in mice during BDENs intervention. (C) The H&E staining of the spleen and kidney, scale bars, 50 μm. Table S1. The primer sequences of qRT-PCR. Table S2 Protein concentration of BDENs.

## Abbreviations

The following abbreviations are used in this manuscript:

MASLD	Metabolic dysfunction-associated steatotic liver disease
BDENs	Broccoli-derived exosome-like nanoparticles
HFD	High-fat diet
miRNA	microRNAs
ALT	Alanine aminotransferase
AST	Aspartate aminotransferase
IL-6	Interleukin-6
TNF-α	TNF-α
IL-1β	Interleukin-1 beta
FDA	U.S. Food and Drug Administration
MASH	Metabolic dysfunction–associated steatohepatitis
FDENs	Food-derived exosome-like nanoparticles
DLS	Dynamic light scattering
TEM	Transmission electron microscope
SPF	Specific pathogen free
Sub	Subcutaneous fat
EP	Epididymal fat
HDL	High-density lipoprotein
LDL	Low-density lipoprotein
IHC	Immunohistochemistry
qRT-PCR	Quantitative Real-Time Polymerase Chain Reaction
BP	Biological processes
CC	Cellular components
MF	Molecular functions
PLS-DA	Partial Least Squares Discrimination Analysis
CDCA	Chenodeoxycholic acid
GCA	Glycocholic acid
DCA	Deoxycholic acid
GaELNs	Garlic-derived exosomal nanoparticles
