# Supplementary material for: Broccoli-Derived Exosome-like Nanoparticles Alleviates Metabolic Dysfunction-Associated Steatotic Liver Disease Through Modulating the Gut–Liver Axis
Source: Nutrients. 2026 Mar 18;18(6):953. doi: 10.3390/nu18060953 (PMC13029203; doi:10.3390/nu18060953)
Supplement: Supplementary file 1 [file nutrients-18-00953-s001.zip › nutrients-4167592-supplementary.pdf]

Broccoli-derived exosome-like nanoparticles alleviates metabolic dysfunction-associated steatotic liver disease through modulating the gut-liver axis

Supplemental Method

Metabolomics

50 mg mouse cecum samples were used to extract metabolites with 400 µL of ex-traction solution (methanol:water = 4:1 (v:v)) containing 0.02 mg/mL of internal standard (L-2-chlorophenylalanine). Samples were cryomilled, cryosonicated and then centrifuged for 15 minutes (4°C, 13,000 g) to collect the supernatant. For LC-MS/MS analyses, the LC-MS/MS analysis of sample was conducted on a Thermo UHPLC-Q Exactive HF-X system equipped with an ACQUITY HSS T3 column (100 mm × 2.1 mm i.d., 1.8 µm; Wa-ters, USA) at Majorbio Bio-Pharm Technology Co. Ltd. (Shanghai, China). The mobile phases consisted of 0.1% formic acid in water:acetonitrile (95:5, v/v) (solvent A) and 0.1% formic acid in acetonitrile:isopropanol:water (47.5:47.5, v/v) (solvent B). The flow rate was 0.40 mL/min and the column temperature was 40°C. The mass spectrometric data were collected using a Thermo UHPLC-Q Exactive HF-X Mass Spectrometer equipped. The optimal conditions were set as followed: source temperature at 425°C; sheath gas flow rate at 50 arb; Aux gas flow rate at 13 arb; ion-spray voltage floating (ISVF) at -3500V in negative mode and 3500V in positive mode, respectively; Normalized collision energy, 20-40-60V rolling for MS/MS. Full MS resolution was 60000, and MS/MS resolu-tion was 7500. Data acquisition was performed with the Data Dependent Acquisition (DDA) mode. The detection was carried out over a mass range of 70-1050 m/z. Each sample was tested with 3 replicates. The pretreatment of LC/MS raw data was performed by Progenesis QI (Waters Corporation Milford, USA) software, and a three-dimensional data matrix in CSV format was exported. Internal standard peaks, as well as any known false positive peaks (including noise, column bleed, and derivatized reagent peaks), were removed from the data matrix, deredundant and peak pooled. At the same time, the metabolites were identified by searching database, and the main databases were the HMDB (http://www.hmdb.ca/), Metlin (https://metlin.scripps.edu/) and Majorbio Data-base. In order to minimise errors arising from sample preparation and instrument instability, the response intensities of the sample's mass spectrometry peaks were normalised using the sum normalisation method, yielding a normalised data matrix. The R package 'ropls' (version 1.6.2) was used to perform principal component analysis (PCA) and orthogonal partial least squares discriminant analysis (OPLS-DA). Based on the variable importance in projection (VIP) obtained from the OPLS-DA model and the p-values from the Student's t-test, metabolites with VIP>1 and $p<0.05$ were selected as significantly differential metabolites. The KEGG database (https://www.kegg.jp/kegg/pathway.html) was used for the metabolic pathway annotation of differential metabolites to identify the pathways in which the differential metabolites are involved.

16S rRNA sequencing

Total microbial genomic DNA was extracted using the TIANamp Stool DNA Kit (Tiangen Biotech (Beijing) Co., Ltd, China) according to manufacturer's instructions. The V3-V4 region of the 16S rRNA genes was subjected to amplification using the 341F (5'-CCTACGGGNGGCWGCAG-3') and

805R (5'-GACTACHVGGGTATCTAATCC-3') primers with TransStart® FastPfu DNA Polymerase (TransGen Biotech, China). Three replicates per sample. The PCR product was extracted from 2% agarose gel and purified using the PCR Clean-Up Kit (YuHua, Shanghai, China) according to manufacturer's in-structions and quantified using Qubit 4.0 (Thermo Fisher Scientific, USA). The sequencing library was generated using the NEXTFLEX Rapid DNA-Seq Kit. The library was sequenced on the Illumina Nextseq2000 platform (Illumina, San Diego, USA). Quality control of raw sequencing reads at both ends was performed using fastp (https://github.com/OpenGene/fastp, version 0.19.6), and assembly was conducted with FLASH (http://www.cbcb.umd.edu/software/flash, version 1.2.11). The DADA2 plugin for QIIME2 was used for denoising. In order to mitigate the influence of sequencing depth on alpha and beta diversity analyses, the number of sequences per sample was standardised to 6,000. The α diversity and β diversity of microbial communities between groups was assessed using Mothur software. Using Principal Coordinate Analysis (PCoA) based on the Bray-Curtis distance algorithm to assess the similarity of microbial com-munity structures among samples. The Lianchuan Biological Cloud Platform https://www.omicstudio.cn/tool/ was used to perform Spearman correlation analysis.

Supplemental Figures

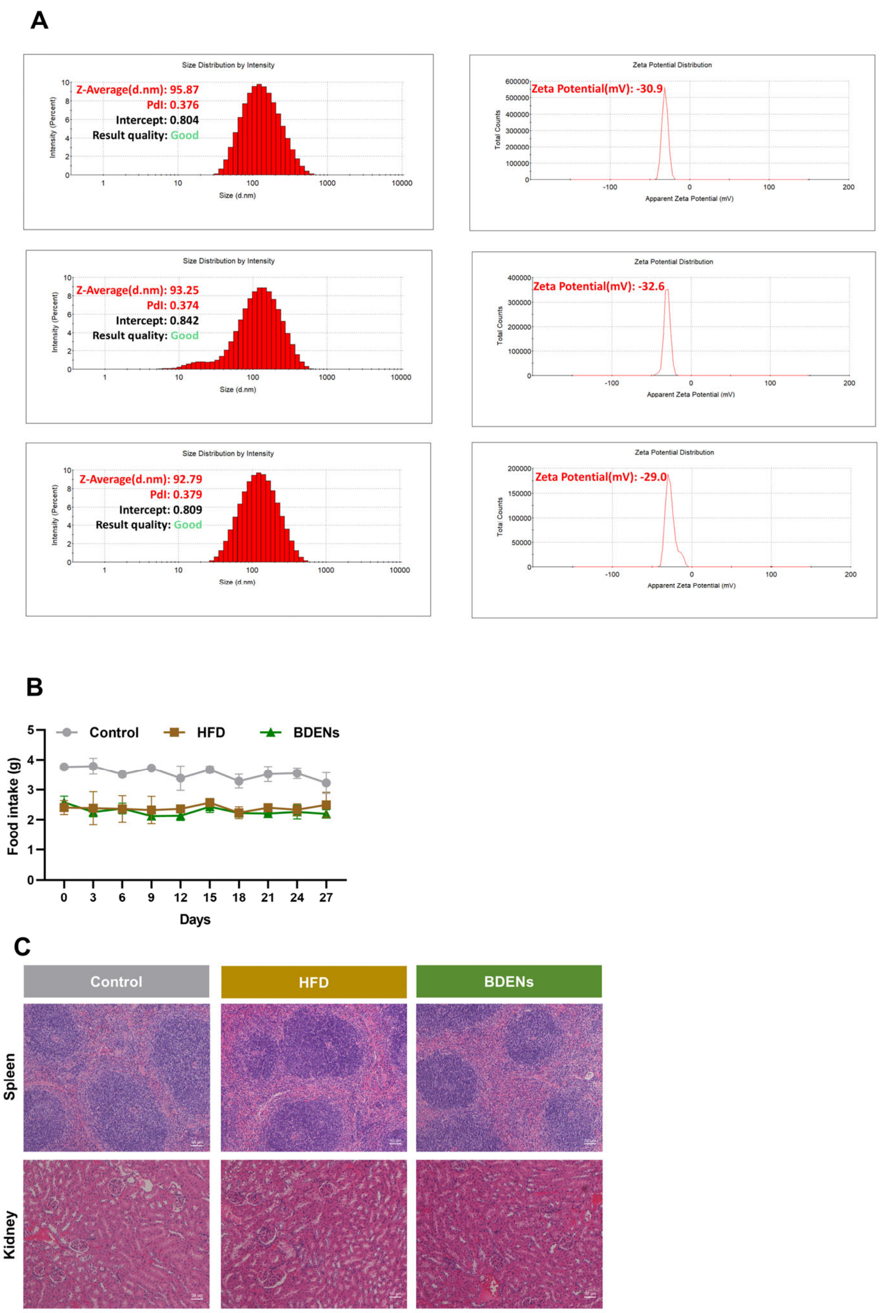

Figure S1. Characterization of BDENs and Assessment of BDENs Side Effects. A Size and zeta potential of BDENs detected by DLS. B Changes in food intake in mice during BDENs intervention. C The H&E staining of the spleen and kidney, scale bars, 50 μm.

Supplemental table

Table S1. The primer sequences of qRT-PCR

Gene	Forward primer	Reverse primer
IL-6 (mouse)	TAGTCCTTCCTACCCCAA TTTCC	TTGGTCCTTAGCCACTCC TTC
TNF-α(mouse)	GACGTGGAACTGGCAGA AGAG	TTGGTGGTTTGTGAGTGT GAG
IL-1β(mouse)	GCAACTGTTCCTGAACTC AACT	ATCTTTTGGGGTCCGTCA ACT

Table S2 Protein concentration of BDENs

Sample	Protein concentration (mg/mL)
BDENs1	9.795
BDENs2	10.095
BDENs3	10.104
Average protein concentration	10.00±0.14
